# Lipidomics Profiles and Lipid Metabolite Biomarkers in Serum of Coal Workers’ Pneumoconiosis

**DOI:** 10.3390/toxics10090496

**Published:** 2022-08-26

**Authors:** Zhangjian Chen, Jiaqi Shi, Yi Zhang, Jiahe Zhang, Shuqiang Li, Li Guan, Guang Jia

**Affiliations:** 1 Department of Occupational and Environmental Health Sciences, School of Public Health, Peking University, Beijing 100191, China; 2 Department of Occupational Disease, Peking University Third Hospital, Beijing 100191, China

**Keywords:** coal workers’ pneumoconiosis, lipidomics, biomarkers, epidemiological study, lipid metabolite

## Abstract

As a serious occupational pulmonary fibrosis disease, pneumoconiosis still lacks effective biomarkers. Previous studies suggest that pneumoconiosis may affect the body’s lipid metabolism. The purpose of this study was to explore lipidomics profiles and lipid metabolite biomarkers in the serum of coal workers’ pneumoconiosis (CWP) by a population case-control study. A total of 150 CWP cases and 120 healthy controls from Beijing, China were included. Blood lipids were detected in serum biochemistry. Lipidomics was performed in serum samples for high-throughput detection of lipophilic metabolites. Serum high density lipoprotein cholesterol (HDL-C) decreased significantly in CWP cases. Lipidomics data found 131 differential lipid metabolites between the CWP case and control groups. Further, the top eight most important differential lipid metabolites were screened. They all belonged to differential metabolites of CWP at different stages. However, adjusting for potential confounding factors, only three of them were significantly related to CWP, including acylhexosylceramide (AHEXCER 43:5), diacylglycerol (DG 34:8) and dimethyl-phosphatidylethanolamine (DMPE 36:0|DMPE 18:0_18:0), of which good sensitivity and specificity were proven. The present study demonstrated that lipidomics profiles could change significantly in the serum of CWP patients and that the lipid metabolites represented by AHEXCER, DG and DMPE may be good biomarkers of CWP.

## 1. Introduction

Pneumoconiosis remains one of the most serious global occupational diseases, especially in China and other developing countries, where the working environment of miners is still very severe. Coal worker’s pneumoconiosis (CWP) is the most common type of pneumoconiosis because the number of coal miners is large, and they easily inhale coal mineral dust and suffer from occupational respiratory diseases. The number of global incident cases of CWP has been relatively stable from 1990 to 2019 [1]. China’s annual national occupational disease official report data also demonstrated that over 90% of reported occupational diseases were pneumoconiosis until 2018, among which CWP accounted for more than 50%. Due to financial compensation and strict diagnostic standards and limited occupational health monitoring, the actual data on pneumoconiosis in China may be even higher [2]. Therefore, the world is concerned about the prevention and treatment of pneumoconiosis [3], particularly in China. China puts forward the Healthy China 2030 Outline and attaches great importance to the development of occupational health, which is represented by the action of pneumoconiosis. It is imperative to carry out scientific research on pneumoconiosis.

Pneumoconiosis is a lung disease with pulmonary fibrosis as the main pathological manifestation due to exposure to dust, especially inorganic dust such as silica [4,5,6]. Previous studies suggest that pneumoconiosis may affect the body’s lipid metabolism, especially lipid peroxidation, which may play a significant role in the pathogenesis of pneumoconiosis [7,8]. The activation of the oxidation system, such as reactive oxygen species (ROS) generation from phagocytes, is the basic physiological process of dust particle-induced lung injury [9,10,11,12]. The exhaled volatile organic compounds (VOCs) derived from lipid peroxidation were used to develop a breath test for pneumoconiosis [13]. However, the effect of pneumoconiosis on lipid metabolism, especially lipid metabolites, still needs more systematic and comprehensive research.

Lipidomics can measure thousands of lipid metabolites with high throughput. Lipids are important components of cell membranes and signaling molecules, and participate in energy storage. With the technological progress of chromatography-mass spectrometry, lipidomics has been widely used to systematically study lipid metabolic changes in biological systems [14,15]. Meanwhile, an untargeted lipidomics platform was jointly developed by international research teams from Japan, China and the United States, accelerating lipidomics research [16]. The method of metabolomics is also applicable in lipidomics, and both are effective tools for development of biomarkers [17,18]. So far, there is still a lack of objective biomarkers to assist in the diagnosis of pneumoconiosis, especially for early health effects. Exploring biomarkers for occupational health monitoring and early diagnosis will contribute to the prevention of pneumoconiosis.

The present study aimed to investigate the perturbation of serum lipidomics profiles in relation to coal worker’s pneumoconiosis (CWP) and explore lipid metabolites as potential biomarkers of CWP. We conducted a case–control study and analyzed the differential metabolome profiles in serum between CWP patients and healthy controls using untargeted lipidomics in high-performance liquid chromatography-mass spectrometry (HPLC-MS). Then, potential serum biomarkers for CWP were screened from differential lipid metabolites through a series of bioinformatics analyses.

## 2. Materials and Methods

### 2.1. Study Subjects

This case–control study included 150 CWP cases and 120 healthy controls from Beijing, China. The cases were recruited from occupational disease hospitals in Beijing and they were all CWP patients that have been clearly diagnosed according to the Chinese occupational disease diagnostic criteria. These CWP patients covered stage 1, stage 2 and stage 3. Healthy controls were recruited from an authoritative health examination institution in Beijing and none of them had occupational exposure to dust. Subjects with a recent history of lung cancer and various other serious respiratory diseases were excluded. The number of subjects with non-respiratory chronic diseases was limited as much as possible. Chronic diseases involved in the study subjects included hypertension, diabetes, coronary heart disease and pulmonary heart disease. Subjects with one or more of these chronic diseases would be judged to have chronic diseases. All subjects obtained informed consent. A detailed self-designed questionnaire was also sent to the research subjects to collect demographic information, including age, smoking, drinking and occupational history. The Peking University Third Hospital Medical Science Research Ethics Committee has approved this study (Approval number: M2024504 and date of approval: 3 November 2021).

### 2.2. Serum Sample Collection

At first, 2 mL of fasting venous blood was collected from each subject in the morning by professional nurses. Then, the blood was allowed to stand for 2–4 h at room temperature. Serum samples were obtained in a cryotube after blood centrifugation (3000 rpm) for 7 min. Finally, the serum samples were inactivated in liquid nitrogen as soon as possible or stored in a refrigerator at −80 °C and not thawed until before use.

### 2.3. Serum Biochemical Lipids Measurement

Routine blood lipid levels were measured by serum biochemisty. Serum biochemical lipids included total cholesterol (T-CHO), triglycerides (TG), high density lipoprotein cholesterol (HDL-C) and low density lipoprotein cholesterol (LDL-C). The measurement of these four serum biochemical lipids were performed by technicians in the laboratory department of the Peking University Third Hospital using an automatic chemistry analyzer.

### 2.4. Serum Sample Preparation of Lipidomics

After the serum was thawed at 4 °C, 100 μL of sample was added to 400 μL of chloroform/methanol (2:1) solution, which was precooled at 4 °C. After thorough mixing and shaking for 5 min, it was centrifuged at 14,000× *g* for 15 min. The solution should have obvious stratification. Then, the lower layer was taken out separately and vacuum freeze-dried for 4 h. The solution was redissolved in 100 μL chloroform/methanol (1:1) solution, and 300 μL diluent (50% isopropanol, 25% acetonitrile, 25% water) was added to dilute the solution. The final 5 μL sample was used for testing on the machine. Meanwhile, quality control (Qc) samples were made for technical repetition by mixing 5 μL of each final sample. Each 5 μL Qc sample was tested at the beginning of the experiment or after analyzing 10 samples.

### 2.5. Detection of Lipidomics in HPLC-MS 

Untargeted lipidomics was performed on a high-performance liquid chromatography-mass spectrometry (HPLC-MS) system. A high-performance liquid chromatography system (UHPLC-Ultimate 3000, Thermo, Waltham, MA, USA) was used for chromatographic separation. The chromatographic column (150 × 2.1 mm; 3 μm; InertSustain C18 HP, GL Sciences, Tokyo, Japan) was equipped with a guard column (10 × 2.1 mm; 3 μm; InertSustain C18, GL Sciences, Tokyo, Japan). The gradient elution time was 25 min. Mobile phase A included 10 mM ammonium formate, 0.1% formic acid, 60% acetonitrile, and 40% water. The mobile phase B included 10 mM ammonium formate, 0.1% formic acid, 90% isopropanol, and 10% acetonitrile. The autosampler temperature was 12 °C, and the injection volume was 10 μL for positive ions and 15 μL for negative ions.

A quadrupole-electrostatic field orbitrap mass spectrometer (Q-Exactive HF, Thermo, Waltham, MA, USA) was used for MS detection. Both the primary mass spectra (MS1) and secondary mass spectra (MS2) were collected. The acquisition mode was data-dependent acquisition. Information on all metabolites within the range of 200–1200 *m*/*z* was collected in MS1 with a resolution of 30,000. Then, MS2 was acquired from the top ten strongest peaks in the MS1. The dynamic collision energy was set to 15, 30 and 45 with a resolution of 15,000. Other MS parameters: the automatic gain control target (AGT) collected by the MS1: 5 × 10^6^; maximum injection time: 20 ms; AGT collected by the MS2: 1 × 10^5^, the maximum injection time: 100 ms; the spray voltage: 3.3 kV for positive electrospray ionization (ESI+) and 3.0 kV for negative electrospray ionization (ESI−); ion source sheath gas 40, auxiliary gas 10, and blowback gas 2; the heating temperature of the ion source gas: 300 °C; the temperature of the ion transfer tube: 320 °C; dynamic exclusion was set to 10 s.

### 2.6. Annotation and Identification of Lipidomics Data

Data preprocessing was conducted by importing the raw MS data into MS-DIAL software v3.6. After converting raw MS data into the standard file format of.abf using the Reifycs ABF converter, MS-DIAL software was used for feature detection, peak alignment, spectrum deconvolution, noise filtering, retention time correction, baseline correction and metabolite identification. The lipid metabolites were annotated and identified through the LipidBlast-based in silico spectra database of MS-DIAL software. The error ranges of MS1 and MS2 were set as 0.01 and 0.05 Da, respectively. The cutoff of identification score was set to 70%.

### 2.7. Analysis of Lipidomics Data

Analysis of lipidomics data was performed by SIMCA15.0.2 software (Sartorius Stedim Data Analytics AB, Umea, Malmö, Sweden). Principal component analysis (PCA) and orthogonal partial least squares discriminant analysis (OPLS-DA) were conducted for multivariate statistics to show the lipidomics profiles. The stability of the OPLS-DA model was verified by permutation tests. Student’s *t* test (data normally distributed) or the Mann-Whitney U test (data not normally distributed) was also conducted for univariate statistics. The differential lipid metabolites between different groups were confirmed by both multivariate and univariate statistics. The false discovery rate (FDR) adjusted *p* < 0.05, |log_2_ fold change (FC)| > 0.25 and the variable importance in the projection (VIP) value > 1 were regarded as the criterion for differential lipid metabolites.

### 2.8. Biomarker Screening

The serum biomarkers were screened from these differential lipid metabolites. Three machine learning methods were applied to rank and find more important differential metabolites. All of them including random forest (RF), support vector machines (SVM) and boruta can be used for dimensionality reduction and screening biomarkers. The top three differential metabolites selected by the three of them, respectively, were combined as potential biomarkers. The potential biomarkers were further verified by multiple logistic regression analysis to adjust for confounding factors. At last, the sensitivity and specificity of biomarkers were evaluated by drawing receiver operating characteristic (ROC) curves.

### 2.9. Statistical Analysis

Methods of statistical analysis for lipidomics data are described above. Basic statistical analysis was conducted in SPSS 20.0. Data are described as the means ± SD or specific number/percentage. For continuous variables, Student’s *t* test (data normally distributed) or the Mann-Whitney U test (data not normally distributed) was applied to evaluate the differences between two independent groups, and one-way variance (ANOVA) with the LSD test was applied for multigroup comparisons. For categorical variables, Pearson’s χ^2^ test was used to compare the rates. Statistical significance was determined when the *p* value was less than 0.05.

## 3. Results

### 3.1. The Characteristics of Subjects

A total of 150 coal worker’s pneumoconiosis (CWP) patients at different stages were included in the case group. The CWP cases at stage 1, stage 2 and stage 3 accounted for 94 (62.7%), 47 (31.3%), and 9 (6.0%), respectively. They all had a clear history of occupational exposure to coal mines, and the mean ± SD of working age was 24.70 ± 8.48 years. Moreover, 19 (12.7%), 32 (21.3%), and 56 (37.3%) cases had complications of tuberculosis, COPD and chronic bronchitis, respectively. In addition, 39 (26.0%) cases had no complications, and four (2.7%) cases had two or more complications. A total of 120 controls with no history of dust occupational exposure were included. As shown in Table 1, significant differences between the CWP case and control groups existed in age, smoking status and chronic diseases (*p* < 0.05). However, no difference existed in drinking status. The average age and the rates of smoking and chronic disease in the case group were higher than those in the control group (*p* < 0.05).

### 3.2. Different Concentrations of Serum Biochemical Lipids between the CWP Case and Control Groups

**Figure 1:**
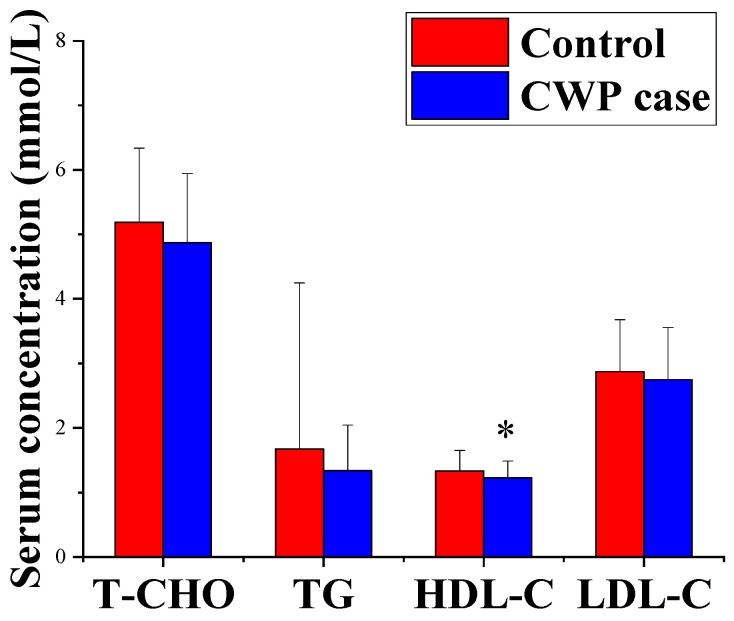
Different concentrations of serum biochemical lipids between the CWP case and control groups. The concentration of serum HDL-C decreased significantly in the CWP case group. * *p* < 0.05, significant difference compared with the control group.

As shown in Figure 1, the concentration of serum HDL-C decreased significantly in the CWP case group compared with the control group (*p* < 0.05). Although there were decreasing trends in serum TCHO, LDL-C and TG, they were not statistically significant (*p* > 0.05). After adjusting for confounding factors, including age, chronic diseases, smoking and drinking, the same results were still maintained. The results indicated that CWP may increase the consumption of lipids, especially HDL-C, resulting in the decreased concentrations of serum biochemical lipids.

### 3.3. Different Lipidomics Profiles between the CWP Case and Control Groups

**Figure 2:**
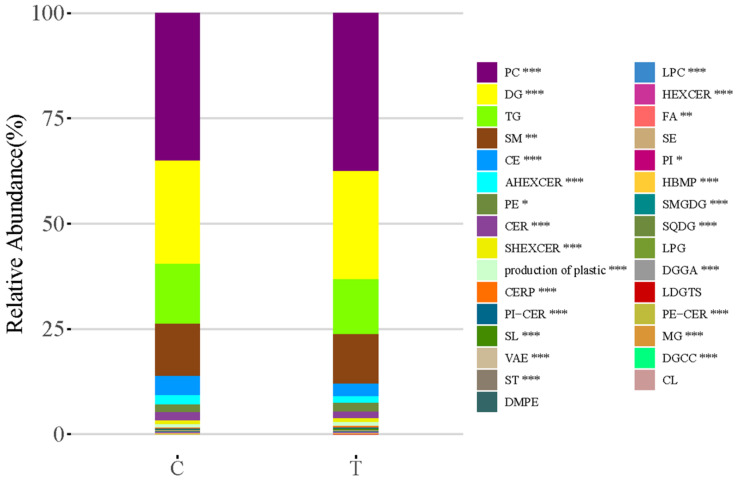
Comparison of the relative abundance of lipid metabolites in subclasses between the CWP case (T) and control (C) groups. The classification of lipid metabolites was identified in serum samples. The relative abundance of lipid metabolites in various subclasses was shown in the statistical stacking histogram. PC, phosphatidylcholine; DG, diacylglycerol; TG, triacylglycerol; SM, sphingomyelin; CE, cholesteryl ester; AHEXCER, acylhexosylceramide; PE, phosphatidylethanolamine; CER, ceramide; SHEXCER, sulfatide; CERP, ceramide 1-phosphates; PI-CER, ceramide phosphatidylinositol; SL, sulfonolipid; VAE, Vitamin A fatty acid ester; ST, sterols; DMPE, dimethyl-phosphatidylethanolamine; LPC, lysophophatidylcholine; HEXCER, hexosylceramide; FA, free fatty acid; SE, sterol ester; PI, phosphatidylinositol; HBMP, hemibismonoacylglycerophosphate; SMGDG, semino monogalactosyldiacylglycerol; SQDG, sulfoquinovosyl diacylglycerol; LPG, lysophosphatidylglycerol; DGGA, diacylglyceryl glucuronide; LDGTS, lysodiacylglyceryl trimethylhomoserine; PE-CER, ceramide phosphoethanolamine; MG, monoacylglycerol; DGCC, diacylglyceryl-3-O-carboxyhydroxymethylcholine; CL, cardiolipin. Significant difference compared to the control group (* *p* < 0.05, ** *p* < 0.01, *** *p* < 0.001).

**Figure 3:**
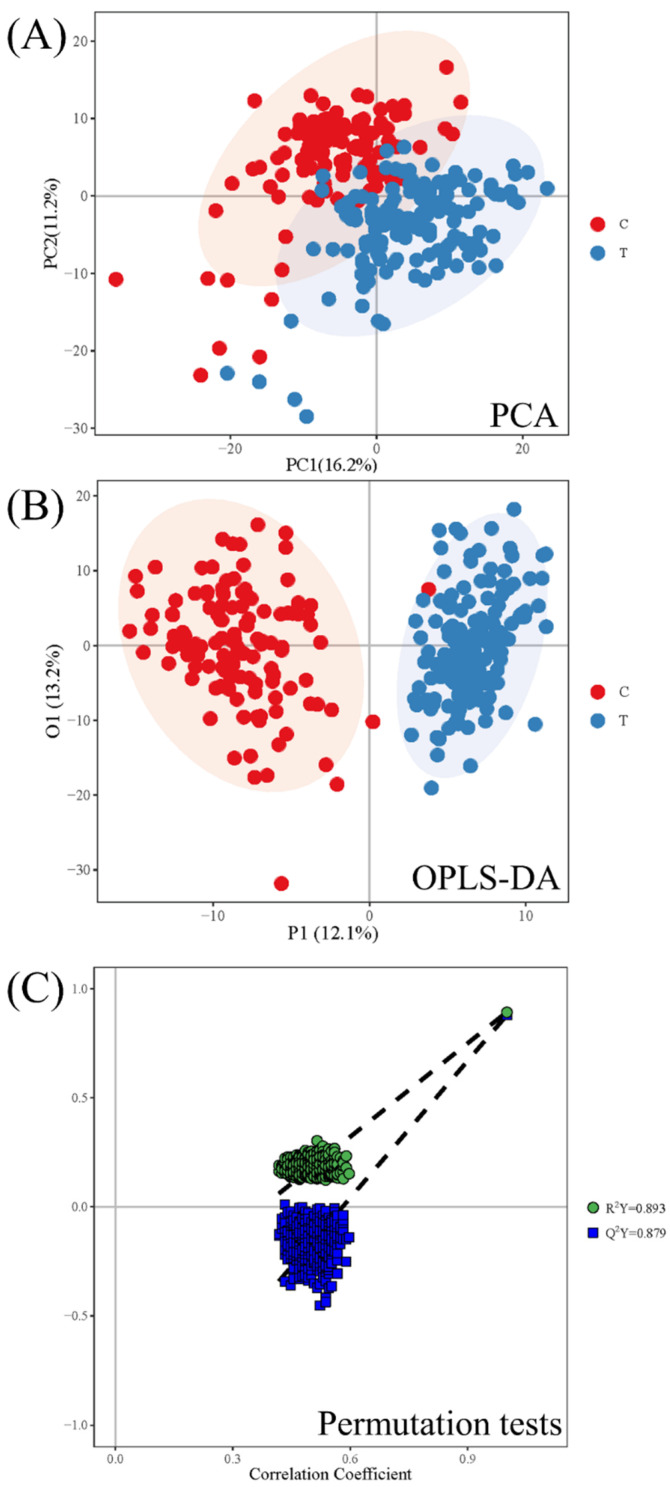
Comparison of the serum lipidomics profiles between the CWP case (T) and control (C) groups. Red dots represent controls and blue dots represent cases. Multivariate statistics were conducted by the PCA model and OPLS-DA model for total lipid metabolites. There was an obvious separation trend between the case group and the control group. Therefore, significant overall differences in lipidomics profiles between the case and control groups were observed in PCA (**A**) and OPLS-DA (**B**) plots. A good OPLS-DA model was verified by the permutation test (**C**).

The difference in lipidomics profiles between the CWP case and control groups was analyzed. A total of 505 lipid metabolites were identified from the MS data. The classification of these lipid metabolites and the comparison of relative abundance in subclasses between the CWP case and control groups are shown in Figure 2. The main lipid metabolite subclasses in the serum samples included phosphatidylcholine (PC), diacylglycerol (DG), triacylglycerol (TG), sphingomyelin (SM), etc. The relative abundance of lipid metabolites in different subclasses was significantly different in the two groups, which suggested that compared with healthy controls, the lipid metabolite profile in the serum of CWP patients changed significantly. Meanwhile, significant overall differences in lipidomics profiles between the two groups were observed in principal component analysis (PCA) (Figure 3A) and orthogonal partial least squares discriminant analysis (OPLS-DA) (Figure 3B). In both multivariate models, all lipid metabolites identified in the lipidomics assay were included in the models for analysis, thus accounting for differences in the overall characteristics of lipidomics profiles between the two groups. A good OPLS-DA model was verified by the permutation test (Figure 3C).

### 3.4. Differential Lipid Metabolites between the CASE and Control Groups

**Figure 4:**
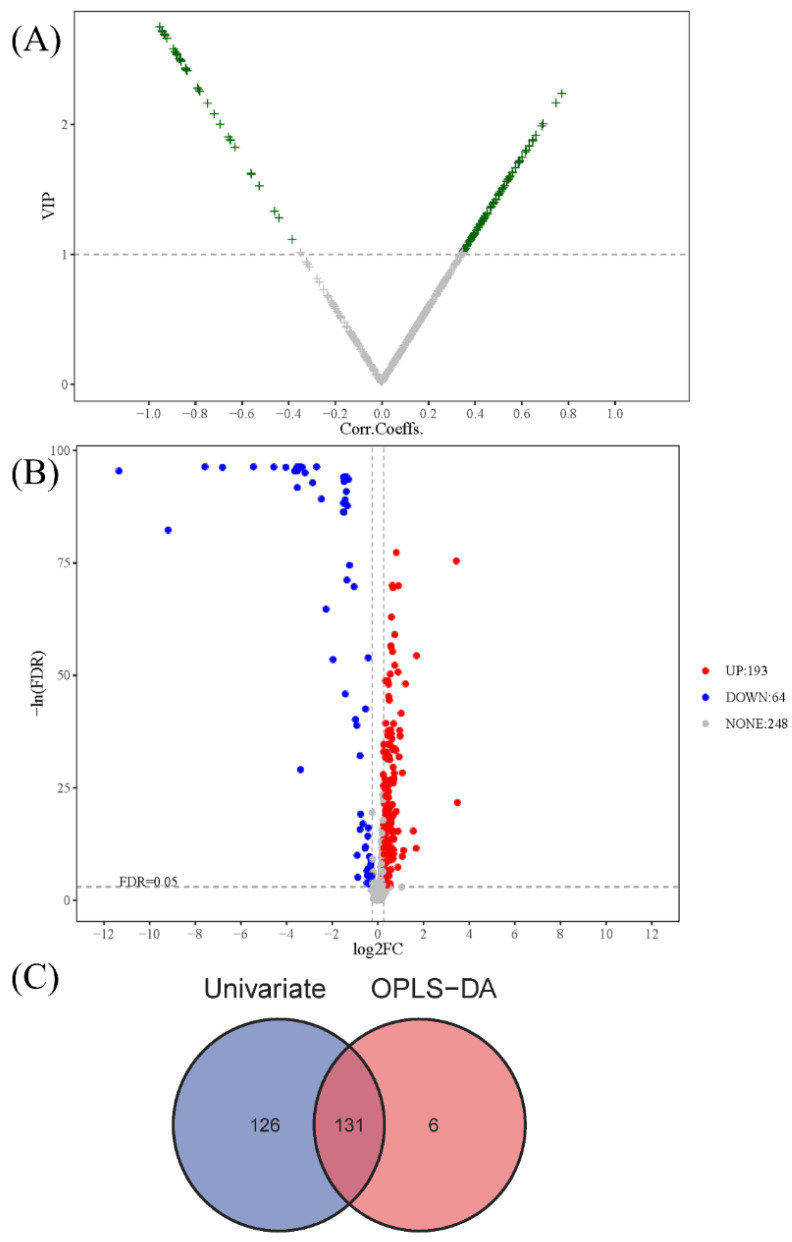
Identification of differential lipid metabolites between the control group and CWP case group. The differential lipid metabolites were distinguished by multivariate statistics (**A**) and univariate statistics (**B**). To distinguish differential metabolites, the selection criterion for multivariate statistics was that the VIP (variable important in projection) value > 1 and for univariate statistics was that FDR adjusted *p* < 0.05 and log_2_FC (fold change) > 0.25. Finally, the differential lipid metabolites were confirmed by overlap of multivariate statistics (OPLS-DA) and univariate statistics in the Venn diagram (**C**).

**Figure 5:**
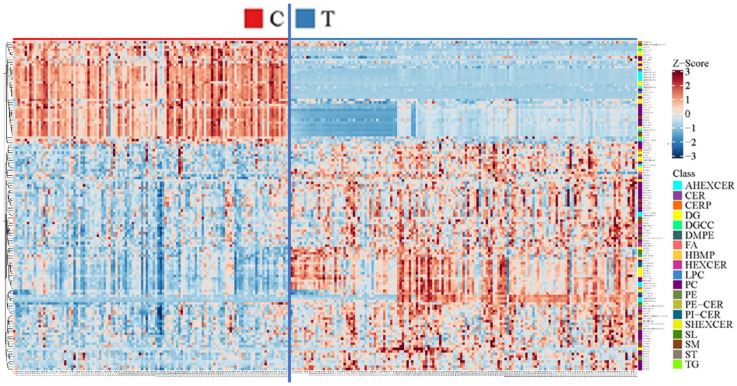
The standard score (Z score) map of differential lipid metabolites between the control group (C) and coal worker’s pneumoconiosis (CWP) case group (T). The Z score was calculated to show the relative abundance of the differential lipid metabolites. The 131 differential lipid metabolites were the overlap of multivariate and univariate statistics.

On the premise of identifying differences in the overall characteristics of lipidomics profiles between CWP cases and controls, specific differential lipid metabolites between the two groups were further analyzed. As a result, a total of 131 differential lipid metabolites were distinguished between the case and control groups. The differential lipid metabolites were analyzed by multivariate statistics (Figure 4A) and univariate statistics (Figure 4B), of which the overlapping part was regarded as the final differential lipid metabolites (Figure 4C) and was used in the subsequent analysis of potential biomarkers. The information on these 131 differential lipid metabolites is displayed in Table S1 (Supplementary file). The relative abundance of these differential metabolites was standardized and clustered, and the heatmap showed that their distribution in the control group and the case group was indeed different (Figure 5). Among these 131 differential metabolites, the relative abundance of 90 metabolites (68.7%) in the CWP case group increased, and the other 41 metabolites (31.3%) decreased.

### 3.5. Screening of Potential Biomarkers for CWP

**Figure 6:**
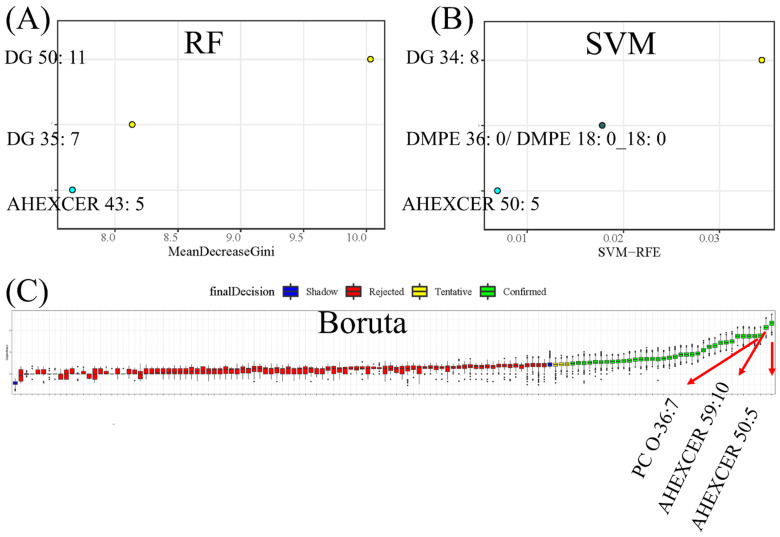
Screening potential biomarkers for coal worker’s pneumoconiosis (CWP) from differential lipid metabolites in serum. Three machine learning methods were used to rank and find more important differential lipid metabolites as potential biomarkers. The top three metabolites selected by RF (**A**), SVM (**B**) and boruta (**C**) were combined. Finally, eight differential lipid metabolites were screened as potential biomarkers, including DG 50:11, DG 35:7, AHEXCER 43:5, DG 34:8, DMPE 36:0|DMPE 18:0_18:0, AHEXCER 50:5, AHEXCER 59:10 and PC O-36:7. RF: random forest; SVM: support vector machine; AHEXCER, acylhexosylceramide; DG, diacylglycerol; PC O-, alkylacyl phosphatidylcholine; DMPE, dimethyl-phosphatidylethanolamine.

**Figure 7:**
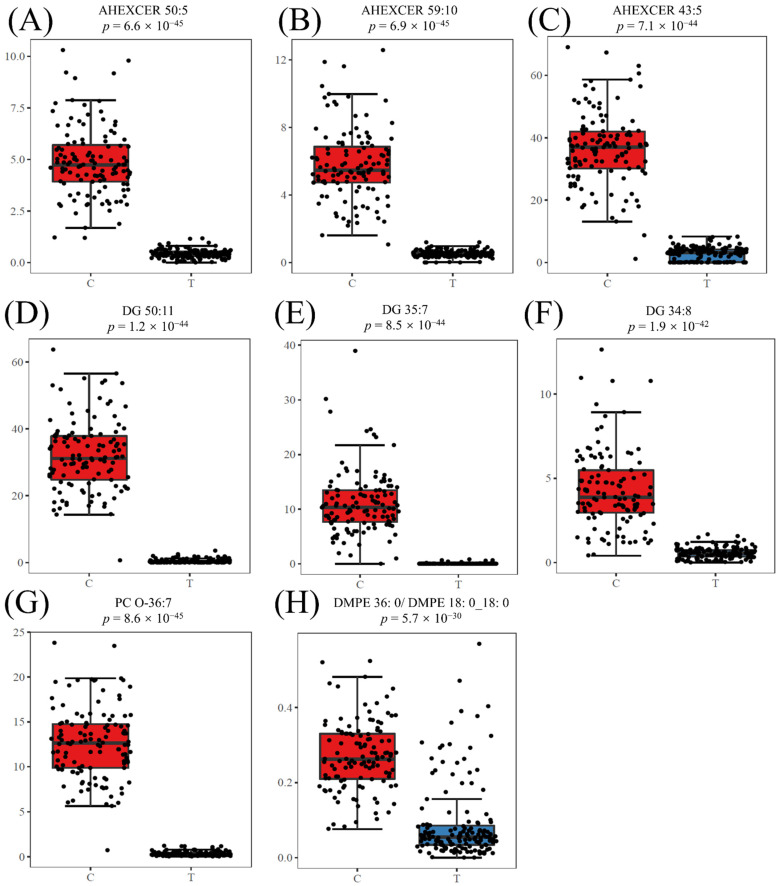
Comparison of the relative abundance of potential biomarkers between the CWP case (T) and control (C) groups. The relative abundances of the eight potential biomarkers of CWP all decreased significantly in the case group (**A**–**H**). The scattered black dots represent one sample data in the case and control groups.

The potential biomarkers for CWP were screened from differential lipid metabolites. Three machine learning (ML) methods were used to rank and find more important differential lipid metabolites as potential biomarkers. The top three metabolites screened by RF were DG 50:11, DG 35:7 and acylhexosylceramide (AHEXCER) 43:5 (Figure 6A). The top three metabolites selected by SVM were DG 34:8, dimethyl-phosphatidylethanolamine (DMPE) 36:0|DMPE 18:0_18:0 and AHEXCER 50:5 (Figure 6B). Then, the boruta method confirmed 36 differential lipid metabolites, among which AHEXCER 50:5, AHEXCER 59:10 and PC O-36:7 were the top three (Figure 6C). Three ML methods screened the top eight most important lipid metabolites, which were initially considered as potential biomarkers for CWP. The relative abundances of these eight potential biomarkers of CWP all decreased significantly in the case group (Figure 7). However, only three lipid metabolites remained significantly related to CWP after adjusting for age, chronic diseases, smoking and drinking. They were AHEXCER 43:5, DG 34:8 and DMPE 36:0|DMPE 18:0_18:0.

### 3.6. Effect of CWP Stage on the Biomarker Screening

**Figure 8:**
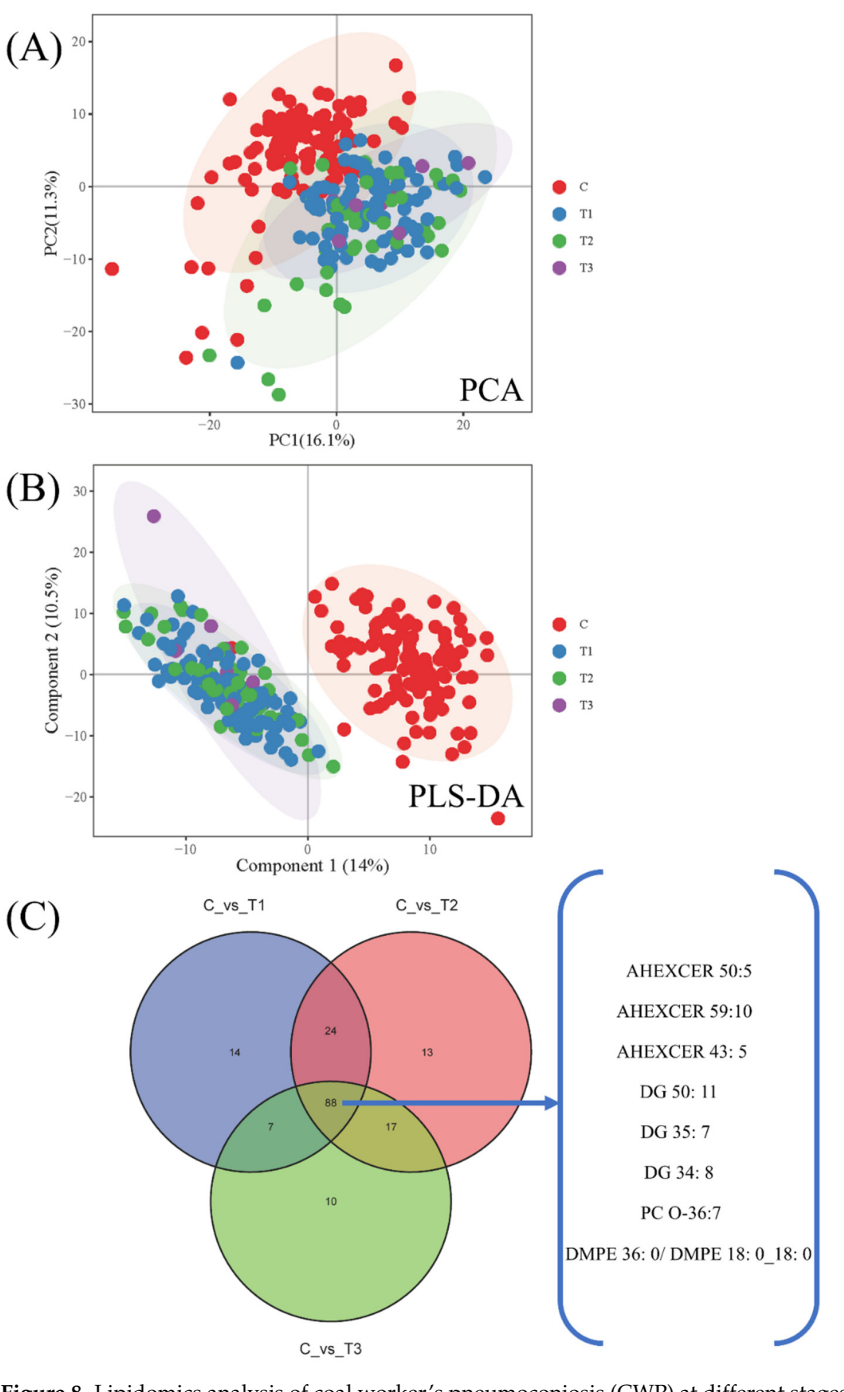
Lipidomics analysis of coal worker’s pneumoconiosis (CWP) at different stages. Significant differences in lipidomics profiles between the CWP case at stage 1 (T1), stage 2 (T2), stage 3 (T3) and the control (C) were observed in PCA (**A**) and PLS-DA (**B**) plots. The distribution of differential lipid metabolites in different groups is also shown in the Venn diagram (**C**). The eight potential biomarkers for CWP were all covered in the intersection of three CWP stages.

**Figure 9:**
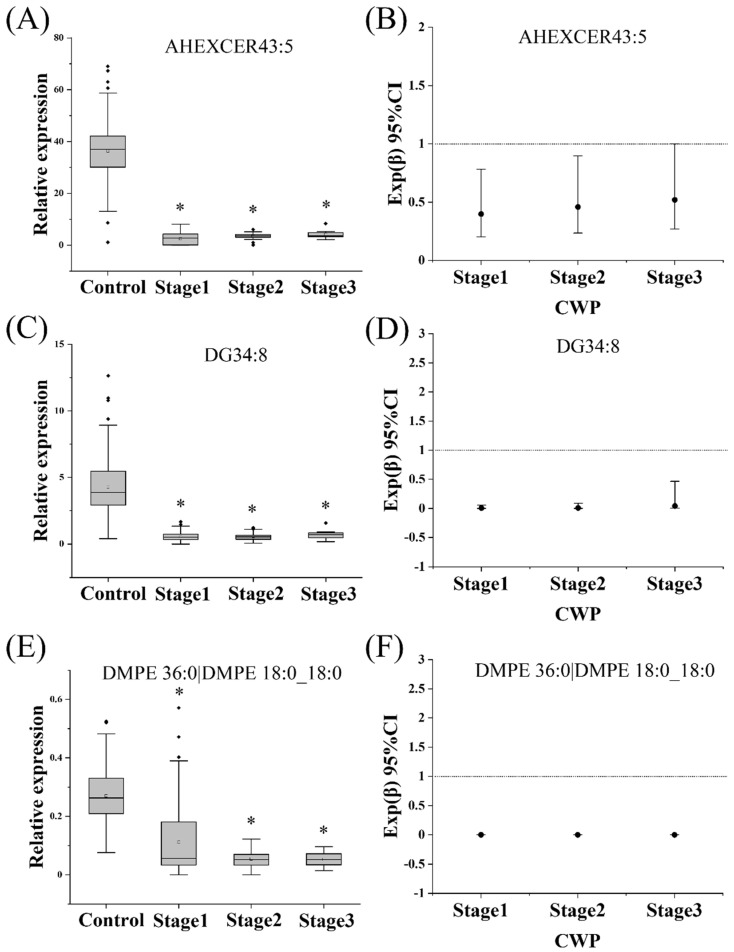
The relationship between the potential biomarkers and coal worker’s pneumoconiosis (CWP) at different stages. Only three potential biomarkers, AHEXCER 43:5, DG 34:8 and DMPE 36:0|DMPE 18:0_18:0, were significantly related to CWP at different stages after adjusting for age, smoking, drinking and chronic diseases (**A**–**F**). Significant difference compared to the control group (* *p* < 0.05).

According to the above analysis strategy, the lipidomics differences between CWP at different stage groups and the control group (Stage 1 vs. control, Stage 2 vs. control, and Stage 3 vs. control) were analyzed respectively. The lipidomics profiles of CWP case groups at stage 1, stage 2 and stage 3 were significantly different from that of the control group (Figure 8A,B). The overall differences among the CWP groups at stage 1, stage 2 and stage 3 were relatively small. However, significant differences were observed between the cases and controls. Analysis of differential lipid metabolites showed that the eight potential biomarkers from previous analysis were all covered in the intersection of differential metabolites of CWP at different stages (Figure 8C). The relationship between the potential biomarkers and different stages of CWP was also analyzed by multiple logistic regression analysis. Similarly, only three lipid metabolites, including AHEXCER 43:5, DG 34:8 and DMPE 36:0|DMPE 18:0_18:0, were significantly related to CWPs at different stages after adjusting for age, chronic diseases, smoking and drinking (Figure 9).

### 3.7. Sensitivity and Specificity Analysis of Potential Biomarkers for CWP

**Figure 10:**
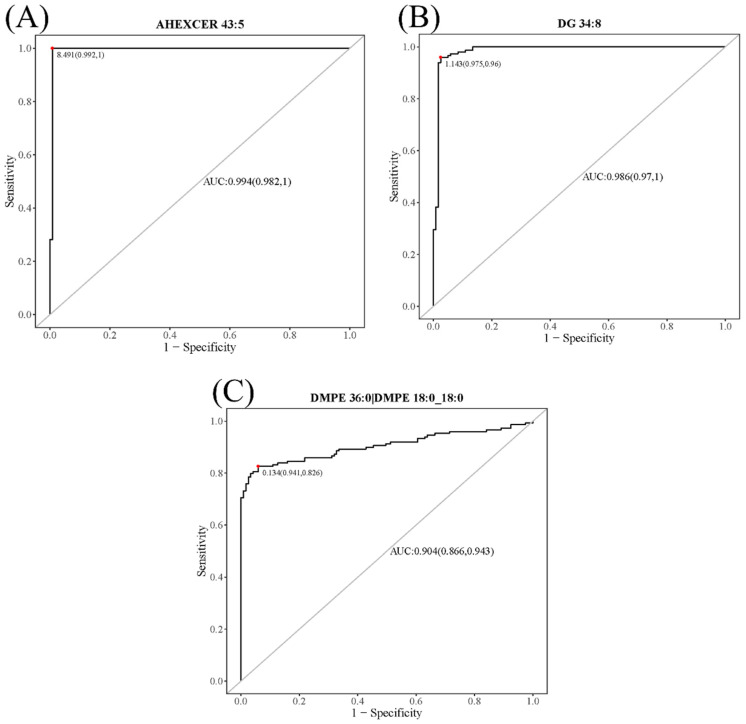
Receiver operating characteristic (ROC) curves of the CWP biomarkers. The area under the ROC curves (AUCs) showed good sensitivity and specificity for AHEXCER 43:5 (**A**), DG 34:8 (**B**) and DMPE 36:0|DMPE 18:0_18:0 (**C**) as biomarkers for CWP.

Receiver operating characteristic (ROC) curves of key potential biomarkers for CWP were drawn. The area under the ROCs (AUC) for AHEXCER 43:5, DG 34:8 and DMPE 36:0|DMPE 18:0_18:0 reached 0.994 (95% CI: 0.982–1.000), 0.986 (95% CI: 0.970–1.000) and 0.904 (95% CI: 0.866–0.943), respectively (Figure 10). Therefore, good sensitivity and specificity were observed for these three differential lipid metabolites.

## 4. Discussion

In the present study, lipidomics was used to explore potential lipid biomarkers in serum for occupational CWP. Untargeted lipidomics is similar to metabolomics and is considered as a very good and high-throughput tool to explore biomarkers [19,20,21,22,23,24,25,26]. Lipidomics focuses more on detecting changes in lipid metabolites, which fits well with the present research. As lipid metabolism disorders are considered to be closely related to pneumoconiosis, there is a lack of systematic research to reveal the different characteristics of lipid metabolism in pneumoconiosis patients and investigate the possibility of using lipid metabolites as biomarkers for CWP. Since lipidomics can study lipid metabolites in the serum of pneumoconiosis patients as a whole, it is feasible to observe the changes in lipid metabolism relatively completely and comprehensively. Meanwhile, the disturbance of metabolites is generally more easily captured, which could be amplified on the basis of gene and protein changes. Therefore, lipidomics is a very suitable method to study lipid metabolism and lipid metabolite-related biomarkers in complex respiratory diseases.

Indeed, significant perturbations of serum biochemical lipids and lipidomics profiles in relation to CWP were observed in this study. Compared with the controls, different lipid metabolite spectra existed not only in the general CWP cases but also in CWP cases at different stages (stage 1, stage 2 and stage 3). Considering that the difference between case groups at different stages (stage 1 vs. stage 2 vs. stage 3) was relatively small, the analysis of differential lipid metabolites was mainly conducted by comparison between the general case and control groups. As a result, a total of 131 differential lipid metabolites were identified between the control group and the CWP case group. The concentration of serum HDL-C also decreased significantly in the CWP case group compared with the control group. This indicated that pneumoconiosis may be related to lipid metabolism disorders. Some previous studies supported this conclusion. The role of lipid metabolism, especially lipid peroxidation, in pneumoconiosis and other respiratory diseases has attracted attention [27,28]. Exposure to silica dust could induce the formation of foam cells in alveolar macrophages (AMs) by lipid accumulation, which increased with the severity of pneumoconiosis and was most apparent in patients at stage 3 [7]. Further cell experiments using NR8383 macrophages also confirmed that combined treatments of SiO_2_ and oxidized LDL (ox-LDL) increased the number and size of foam cells, driving TGF-β production and fibrotic responses. Kazuhiro et al also revealed that Clara cells played an important role in the foam cell formation of AMs in crystalline silica-exposed mice [29]. Meanwhile, activation of oxidant production by pulmonary phagocytes is one of the basic mechanisms in the etiology of CWP and silicosis, which could cause lipid peroxidation and eventually lead to lung injury and scarring [8]. Therefore, lipid metabolism disturbance should play an important role in the early onset of pneumoconiosis, and lipid metabolites may be important biomarkers for CWP. However, there was no report on the association between pneumoconiosis or silica inhalation and HDL decline, which may be worthy of further study.

In order to achieve the goal of dimension reduction, machine learning (ML) methods were used to rank the importance of differential lipid metabolites between CWP cases and controls. Through three different ML methods, the eight most important lipid metabolites were screened out from the 131 differential lipid metabolites. The ML methods used in the present study were RF, SVM and boruta. As the three of them have advantages and disadvantages when used for dimensionality reduction analysis, the top three lipid metabolites selected by RF, SVM and boruta were combined. Only one lipid metabolite of acylhexosylceramide 50:5 (AHEXCER 50:5) was simultaneously selected by SVM and boruta. The three ML methods did not screen out other overlapping metabolites. Therefore, eight differential lipid metabolites were screened as potential biomarkers of CWP, represented by acylhexosylceramide (AHEXCER), diacylglycerol (DG), alkylacyl phosphatidylcholine (PC O-), and dimethyl-phosphatidylethanolamine (DMPE).

Among these differential lipid metabolites between CWP cases and controls, the importance of AHEXCER and DG ranked relatively high, even though they may be several similar lipids with different lengths of carbon chains. For example, AHEXCER 43:5, AHEXCER 50:5 and AHEXCER 59:10 all belonged to potential biomarkers of CWP. Acylhexosylceramide (AHEXCER) is a kind of sphingolipid (SP), and the main class is neutral glycosphingolipids (SP05). Sphingolipids are important lipids due to their functions involved in membrane components, cellular signaling and various diseases [30]. SP levels could be altered in many human diseases, such as cancer [31,32], diabetes [33], and cardiovascular diseases [34,35]. More importantly, previous studies have shown that sphingolipids, especially ceramide, play an important role in the pathogenesis of cystic fibrosis [36,37], which is one of the major lung fibrosis diseases and similar to the main pathological features of pneumoconiosis. Human and animal studies have shown that ceramide could accumulate in the cystic fibrosis lung and induce inflammation and high susceptibility to bacterial infections [38,39]. Some clinical studies have demonstrated that sphingolipids may be a potential new target for therapeutic intervention for cystic fibrosis [40]. However, the structures and metabolism of SPs are complex, even though significant progress has been made in identifying most of them with the improvement of analytical mass spectrometry [41]. Interestingly, acylhexosylceramide, as a biomarker of CWP (AHEXCER 43:5, AHEXCER 50:5 and AHEXCER 59:10), appeared to have very long chain lengths, and its relative expression decreased significantly. The specific changes in sphingolipid metabolism in CWP patients, especially acylhexosylceramide, and the underlying mechanism need to be further studied.

Diacylglycerol (DG), including DG 50:11, DG 35:7 and DG 34:8, also belonged to potential biomarkers of CWP. As an intermediate product of triacylglycerol hydrolysis, DG is a kind of glycerolipid (GL), and the main class is diradylglycerols (GL02). Diacylglycerol (DG) is also often referred to as DAG, which is composed of a glycerol backbone esterified with two fatty acids, including three stereochemical isoforms. DG can be found naturally in various vegetable oils or synthesized by structural modification of conventional fats and oils. Some previous studies have demonstrated that DG could suppress body fat accumulation and decrease postprandial serum levels of triacylglycerol, cholesterol and glucose [42]. In the present study, the relative expression of three DGs as potential biomarkers of CWP all decreased significantly, which may reduce their beneficial effects on health. However, increased levels of DG were also associated with hepatic insulin resistance and nonalcoholic fatty liver disease [43,44,45]. Meanwhile, body mass index (BMI) may also affect the function of the DG [46]. For example, DG may be ineffective in controlling the glucose level in obese individuals. However, the present study lacked relevant personal information, so the relationship between DG and CWP still needs to be confirmed by follow-up research.

The influence of confounding factors must be controlled as much as possible to obtain accurate results in this epidemiological study. Logistic regression analysis was conducted to adjust for confounding factors, and only three potential biomarkers were verified to be significantly related to CWP. These three lipid metabolites were retained as good biomarkers of CWP, namely, AHEXCER 43:5, DG 34:8 and DMPE 36:0|DMPE 18:0_18:0. The types of major lipids did not change, but some lipid metabolites with other length chains were excluded. This indicated that the change in some DGs may be caused by confounding factors, such as age. Indeed, lipid metabolism can play an important role in the aging process, and blood triglyceride levels tend to increase with age in humans [47]. Previous research has shown that the influence of age on diacylglycerol content in muscle is obvious, represented by a lower content of total unsaturated C24:0 DGs in the muscle of aged people [48]. The average age of the CWP group was older than that of the control group, which may be another reason for the decrease in some DGs in the CWP group. For the remaining three lipid metabolites after adjusting for confounding factors, the sensitivity and specificity were evaluated to be good by calculating the area under the ROC curves. The series of statistical analysis methods have good effectiveness in exploring good biomarkers of CWP, which may also apply to other respiratory diseases.

In this study, lipidomics and human samples were used to study the serum biomarkers of CWP. In view of the lack of objective biomarkers of pneumoconiosis and its close association with lipid metabolism, the research has strong practicability. In particular, the combination of untargeted lipidomics and machine learning methods is conducive to the discovery of good biomarkers by improving the comprehensiveness and accuracy. However, there were also some limitations in this study. The primary limitation is the control of confounding factors. Even if the influence of confounding factors was controlled by statistics, the incomplete match between the case and control groups may still affect the results. In particular, the effects of age and chronic diseases on metabolism may be complex and may not be completely controlled by simple continuous variables or categorical variables. However, this is indeed a difficult problem to overcome in the study of pneumoconiosis because the age of the cases is generally too old and complicated with chronic diseases. We tend to study these factors as a whole, which belongs to the current situation of pneumoconiosis, rather than simply studying the harm of dust. The second limitation is the difference between metabolic perturbations and clinically significant changes in diseases. The main body of untargeted lipidomics detection is still small molecule lipid metabolites. Their biological significance is different from serum lipids detected by routine blood biochemistry, so the clinical significance needs to be further explored. Finally, the present study needs further validation to increase the strength of evidence. Using targeted lipidomics in another similar population, preferably a population with a larger number of samples, will help to increase the credibility of the conclusion.

## 5. Conclusions

In conclusion, the serum lipidomics profile of CWP cases was significantly different from the healthy controls. A total of 131 differential lipid metabolites were identified, and three of them, AHEXCER 43:5, DG 34:8 and DMPE 36:0|DMPE 18:0_18:0, may be good biomarkers of CWP. However, the biological association between them needs to be further determined. Lipidomics combined with ML methods should be an effective tool for the discovery of biomarkers of respiratory diseases, represented by pneumoconiosis.

## Figures and Tables

**Table 1:** Comparison of characteristics of subjects in the coal worker’s pneumoconiosis (CWP) case and control groups.

Variables	Control Group (*n* = 120)	CWP Case Group (*n* = 150)	*p* Value
Age (years)	56.63 ± 3.03	69.02 ± 9.07	<0.001 *
Smoking *n* (%)			<0.001 *
Yes	63 (52.5)	125 (83.3)	
No	57 (47.5)	25 (16.7)	
Dinking *n* (%)			0.934
Yes	69 (57.5)	87 (58.0)	
No	51 (42.5)	63 (42.0)	
Chronic disease *n* (%)			<0.001 *
Yes	51 (42.5)	106 (70.7)	
No	69 (57.5)	44 (29.3)	

* *p* < 0.05, significant difference compared with the control group.

## Data Availability

The data presented in this study are available on request from the corresponding author. The data are not publicly available due to the regulations of the authors’ affiliations.

## Supplementary Materials

The following supporting information can be downloaded at: https://www.mdpi.com/article/10.3390/toxics10090496/s1, Table S1: Differential lipophilic metabolites in serum between the occupational pneumoconiosis group and the control group.
