# Supplementary material for: Lipidomics Profiles and Lipid Metabolite Biomarkers in Serum of Coal Workers’ Pneumoconiosis
Source: Toxics. 2022 Aug 26;10(9):496. doi: 10.3390/toxics10090496 (PMC9500698; doi:10.3390/toxics10090496)
Supplement: Supplementary file 1 [file toxics-10-00496-s001.zip › toxics-1857224-supplementary.pdf]

Table S1. Differential lipophilic metabolites in serum between the occupational pneumoconiosis group and the control group.

No.	Metabolite	Class	P value	FDR	log_2 Fold Change	VIP
1	AHEXCER 40:4	AHEXCER	2.16E-25	2.54E-24	3.248	1.699
2	AHEXCER 41:4	AHEXCER	2.87E-26	3.54E-25	1.496	0.582
3	AHEXCER 41:5	AHEXCER	7.84E-43	2.33E-41	0.413	-1.275
4	AHEXCER 42:5	AHEXCER	2.22E-26	2.81E-25	1.499	0.584
5	AHEXCER 43:3	AHEXCER	1.42E-23	1.49E-22	1.459	0.545
6	AHEXCER 43:5	AHEXCER	7.12E-44	3.58E-42	0.088	-3.514
7	AHEXCER 44:4	AHEXCER	2.93E-09	1.16E-08	1.292	0.369
8	AHEXCER 44:5	AHEXCER	8.49E-08	2.66E-07	1.315	0.395
9	AHEXCER 44:6	AHEXCER	2.45E-44	1.62E-42	0.062	-4.022
10	AHEXCER 45:4	AHEXCER	4.26E-19	3.71E-18	0.512	-0.967
11	AHEXCER 50:5	AHEXCER	6.56E-45	1.45E-42	0.087	-3.524
12	AHEXCER 53:8	AHEXCER	1.09E-34	1.84E-33	10.913	3.448
13	AHEXCER 56:9	AHEXCER	8.31E-44	3.58E-42	0.080	-3.635
14	AHEXCER 59:10	AHEXCER	6.86E-45	1.45E-42	0.095	-3.389
15	CER 30:4	CER	5.33E-18	4.2E-17	1.959	0.970
16	CER 47:11	CER	1.41E-43	5.49E-42	0.111	-3.173
17	CER 50:11	CER	6.05E-10	2.61E-09	1.266	0.340
18	CER 50:6	CER	8.45E-13	4.49E-12	1.527	0.611
19	CERP 28:2	CERP	1.18E-08	4.3E-08	0.641	-0.641
20	CERP 38:2	CERP	3.54E-29	4.7E-28	1.528	0.611
21	CERP 39:2	CERP	3.75E-16	2.56E-15	1.706	0.771
22	DG 23:3	DG	2.96E-32	4.4E-31	1.874	0.906
23	DG 24:3	DG	4.74E-32	6.65E-31	1.602	0.680
24	DG 25:3	DG	7.99E-41	1.83E-39	0.181	-2.466
25	DG 25:4	DG	2.32E-15	1.45E-14	1.920	0.941
26	DG 28:2	DG	1.08E-17	8.25E-17	1.521	0.605
27	DG 34:8	DG	1.88E-42	4.99E-41	0.139	-2.850
28	DG 35:4	DG	1.58E-18	1.3E-17	0.533	-0.907
29	DG 35:7	DG	8.5E-44	3.58E-42	0.000	-11.334
30	DG 38:5	DG	6.51E-08	2.1E-07	2.944	1.558

Table S1. Differential lipophilic metabolites in serum between the occupational pneumoconiosis group and the control group. (**Continued**)

No.	Metabolite	Class	P value	FDR	log_2 Fold Change	VIP
31	DG 39:10	DG	4.32E-15	2.6E-14	1.450	0.536
32	DG 39:2	DG	8.68E-09	3.22E-08	1.506	0.591
33	DG 39:8	DG	1.06E-37	1.92E-36	0.002	-9.176
34	DG 40:6	DG	4.74E-43	1.5E-41	0.366	-1.452
35	DG 41:11	DG	5.39E-21	4.95E-20	1.425	0.511
36	DG 43:8	DG	1.44E-44	1.45E-42	0.157	-2.671
37	DG 44:10	DG	1.01E-40	2.22E-39	0.373	-1.422
38	DG 45:11	DG	5.38E-25	6.04E-24	0.258	-1.956
39	DG 49:12	DG	1.91E-24	2.09E-23	1.684	0.752
40	DG 50:11	DG	1.23E-44	1.45E-42	0.005	-7.553
41	DG 50:13	DG	8.18E-26	9.83E-25	1.579	0.659
42	DG 50:14	DG	9.74E-19	8.33E-18	1.274	0.350
43	DG 51:10	DG	3.62E-25	4.15E-24	0.748	-0.418
44	DG 51:14	DG	4.16E-12	2.06E-11	1.268	0.343
45	DG 52:11	DG	8.08E-16	5.23E-15	1.318	0.399
46	DGCC 40:5	DGCC	5.81E-42	1.47E-40	0.087	-3.520
47	DMPE 36:0|DMPE 18:0_18:0	DMPE	5.71E-30	7.8E-29	0.209	-2.256
48	FA 18:1	FA	3.99E-10	1.74E-09	1.289	0.367
49	HBMP 45:3|HBMP 15:1_15:1_15:1	HBMP	3.83E-13	2.13E-12	1.249	0.320
50	HEXCER 34:1	HEXCER	1.72E-09	6.88E-09	1.292	0.370
51	HEXCER 41:1	HEXCER	1.34E-16	9.43E-16	1.209	0.274
52	HEXCER 43:1	HEXCER	8.55E-13	4.5E-12	1.423	0.509
53	LPC 19:2/0:0	LPC	3.21E-13	1.8E-12	1.647	0.720
54	LPC O-28:7	LPC	3E-44	1.68E-42	0.009	-6.797
55	PC 26:0	PC	6.99E-08	2.23E-07	1.867	0.900
56	PC 29:0	PC	2.85E-34	4.64E-33	0.426	-1.232
57	PC 30:0	PC	2.86E-08	9.68E-08	1.407	0.492
58	PC 30:2	PC	1.02E-19	9.01E-19	2.037	1.027
59	PC 31:0	PC	2.54E-14	1.51E-13	1.606	0.683
60	PC 32:2	PC	6.03E-18	4.69E-17	1.370	0.454

Table S1. Differential lipophilic metabolites in serum between the occupational pneumoconiosis group and the control group. (**Continued**)

No.	Metabolite	Class	P value	FDR	log_2 Fold Change	VIP
61	PC 33:2	PC	1.32E-42	3.72E-41	0.361	-1.470
62	PC 33:3	PC	1.67E-17	1.26E-16	1.360	0.444
63	PC 35:6	PC	1.06E-18	8.92E-18	1.631	0.706
64	PC 36:6	PC	1.8E-15	1.15E-14	0.587	-0.768
65	PC 36:7	PC	2.56E-44	1.62E-42	0.102	-3.294
66	PC 38:5	PC	1.32E-13	7.49E-13	1.189	0.250
67	PC 39:4	PC	3.71E-32	5.36E-31	0.490	-1.030
68	PC 39:9	PC	2.24E-21	2.1E-20	1.406	0.492
69	PC 40:4	PC	6.96E-12	3.38E-11	1.386	0.471
70	PC 41:6	PC	1.8E-44	1.51E-42	0.043	-4.542
71	PC 42:8	PC	8.67E-10	3.62E-09	1.279	0.355
72	PC 43:5	PC	2.15E-15	1.36E-14	1.415	0.501
73	PC 44:11	PC	5.26E-16	3.5E-15	1.745	0.803
74	PC 44:5	PC	1.31E-10	5.87E-10	1.362	0.445
75	PC 44:9	PC	5.04E-18	4.04E-17	1.464	0.549
76	PC O-29:0	PC	5.73E-09	2.18E-08	1.281	0.357
77	PC O-30:1	PC	3.95E-09	1.53E-08	1.299	0.377
78	PC O-31:0	PC	6.53E-23	6.73E-22	1.275	0.351
79	PC O-32:0	PC	1.54E-12	7.84E-12	1.387	0.472
80	PC O-33:4	PC	1.58E-35	2.75E-34	1.764	0.819
81	PC O-34:0	PC	1.09E-10	4.92E-10	1.230	0.299
82	PC O-34:4	PC	3.81E-20	3.43E-19	0.695	-0.524
83	PC O-35:4	PC	2.64E-32	4.04E-31	1.561	0.643
84	PC O-36:4	PC	1.83E-39	3.42E-38	0.361	-1.470
85	PC O-36:7	PC	8.59E-45	1.45E-42	0.023	-5.438
86	PC O-37:6	PC	7.84E-33	1.24E-31	0.395	-1.342
87	PC O-37:7	PC	1.02E-08	3.73E-08	1.219	0.285
88	PC O-38:2	PC	2.18E-11	1.03E-10	1.228	0.296
89	PC O-38:7	PC	1.32E-08	4.77E-08	1.437	0.523
90	PC O-40:7	PC	4.35E-14	2.55E-13	0.096	-3.375

Table S1. Differential lipophilic metabolites in serum between the occupational pneumoconiosis group and the control group. (**Continued**)

No.	Metabolite	Class	P value	FDR	log_2 Fold Change	VIP
91	PC O-41:2|PC O-23:0_18:2	PC	9.54E-09	3.52E-08	1.251	0.323
92	PC O-41:5	PC	7.01E-16	4.6E-15	1.198	0.261
93	PC O-42:8	PC	1.08E-10	4.9E-10	1.373	0.458
94	PC O-43:6	PC	1.27E-16	9.01E-16	1.403	0.488
95	PC O-43:7	PC	4.02E-40	8.11E-39	0.401	-1.317
96	PC O-44:8	PC	6.09E-07	1.78E-06	1.195	0.257
97	PC O-44:9	PC	1.74E-08	6.12E-08	1.285	0.362
98	PC O-45:10	PC	1.79E-39	3.42E-38	0.354	-1.497
99	PC O-45:9	PC	2.27E-40	4.77E-39	0.353	-1.500
100	PC O-46:10	PC	8.37E-11	3.84E-10	11.277	3.495
101	PC O-48:7	PC	1.11E-09	4.6E-09	1.455	0.541
102	PC O-48:8	PC	3.42E-09	1.34E-08	1.509	0.594
103	PE-CER 42:3	PE-CER	1.89E-16	1.31E-15	1.504	0.589
104	PE 34:3	PE	8.78E-14	5.09E-13	2.123	1.086
105	PE 36:5	PE	9.82E-13	5.06E-12	1.601	0.679
106	PE O-37:1|PE O-19:0_18:1	PE	4.43E-09	1.71E-08	1.298	0.377
107	PE O-39:4|PE O-19:0_20:4	PE	4.82E-12	2.36E-11	1.303	0.382
108	PE O-41:4|PE O-19:0_22:4	PE	1.53E-22	1.48E-21	1.393	0.478
109	PI-CER 26:2	PI-CER	4.63E-18	3.77E-17	1.476	0.562
110	PI-CER 31:3	PI-CER	6.96E-23	7.03E-22	1.367	0.451
111	PI-CER 37:7	PI-CER	3.76E-15	2.29E-14	1.348	0.431
112	SHEXCER 38:3	SHEXCER	2.59E-11	1.21E-10	1.389	0.474
113	SL 32:1	SL	4.38E-16	2.95E-15	1.583	0.663
114	SL 36:6	SL	9.05E-24	9.72E-23	1.868	0.902
115	SL 42:6	SL	1.72E-27	2.22E-26	1.680	0.748
116	SM 33:0	SM	1.33E-22	1.31E-21	2.324	1.217
117	SM 34:0	SM	2.64E-15	1.62E-14	1.266	0.340
118	SM 34:1	SM	1.89E-11	9.01E-11	1.281	0.357
119	SM 36:0	SM	9.49E-13	4.94E-12	1.362	0.445
120	SM 36:3	SM	3.68E-17	2.66E-16	1.538	0.621

Table S1. Differential lipophilic metabolites in serum between the occupational pneumoconiosis group and the control group. (**Continued**)

No.	Metabolite	Class	P value	FDR	log_2 Fold Change	VIP
121	SM 36:4	SM	2.94E-10	1.29E-09	1.438	0.524
122	SM 36:6	SM	1.5E-41	3.61E-40	0.387	-1.368
123	SM 39:6	SM	4.25E-43	1.43E-41	0.358	-1.483
124	SM 41:6	SM	3.56E-43	1.29E-41	0.389	-1.362
125	SM 42:2	SM	1.78E-12	9E-12	1.195	0.257
126	SM 43:1	SM	6.78E-13	3.64E-12	1.624	0.699
127	SM 44:4	SM	4.31E-13	2.36E-12	1.495	0.580
128	SM 48:6	SM	2.68E-08	9.15E-08	1.297	0.375
129	SM 48:7	SM	1.38E-09	5.63E-09	1.390	0.475
130	ST 24:1	ST	1.74E-17	1.29E-16	1.390	0.475
131	TG 53:9	TG	1.27E-21	1.21E-20	0.376	-1.410

AHEXCER, Acylhexosylceramide; HEXCER, Hexosylceramide; SHEXCER, Sulfatide; CER, Ceramide; CERP, Ceramide 1-phosphates; DG, diacylglycerol; DGCC, Diacylglyceryl-3-O-carboxyhydroxymethylcholine; FA, Free fatty acid; HBMP, Hemibismonoacylglycerophosphate; LPC, lysophosphatidylcholine; PC, Phosphatidylcholine; PC-O, alkylacyl PC; PE, phosphatidylethanolamine; PE-O, alkylacyl PE; PE-CER, Ceramide phosphoethanolamine; DMPE, dimethyl-phosphatidylethanolamine; PI, phosphatidylinositol; PI-CER, Ceramide phosphatidylinositol; SL, Sulfonolipid; SM, sphingomyelin; ST, Sterols; TG, triacylglycerol.
